# Editorial: Emerging learnings in cell therapy: novel binding domains, universal CAR-T cells, and more

**DOI:** 10.3389/fonc.2024.1404376

**Published:** 2024-04-15

**Authors:** Anand Rotte

**Affiliations:** Department of Clinical and Regulatory Affairs, Arcellx Inc, Redwood City, CA, United States

**Keywords:** CAR-T cells, D-Domains, protein aggregation, Tonic signaling, cell therapy (CT)

This recent decade has seen a dramatic improvement in the potential of immunotherapy and cell therapy as options for the treatment of cancer (1–6). Research interest in cell therapy has increased significantly over the past few years, with a focus on improving efficacy and addressing challenges (7) that has resulted in the introduction of novel target binding domains, dual intracellular signaling domains, T cells redirected for universal cytokine-mediated killing (TRUCKs), and allogeneic cell therapies (8). The current Research Topic, “*Emerging learnings in cell therapy: novel binding domains, universal CAR-T cells, and more*” aimed to attract new innovative research on cell therapy for cancer. Articles published in the Research Topic include two review articles, five preclinical studies, two studies reporting findings from real-world use of CAR-T cells, and one study reporting findings on atypical T-cell receptor (TCR)-T cells.

The review article by Zhang et al. provided a summary of recent developments in CAR-T cell therapy for solid tumors. The authors mainly discussed the strategies developed to increase the efficacy of CAR-T cells in solid tumors through the use of dual-targets, receptor switches, and CARs that are designed to resist the inhibitory signaling molecules in the TME. The second review article by Liu et al. summarized the latest advances and updates from the ASH 2022 annual meeting on anti-CD7 CAR-T cells and their application for treatment of acute myeloid leukemia and T-Lymphocyte Leukemia and Lymphoma.

The importance of intracellular costimulatory domain (4-1-BB) in activating non-canonical NF-κB pathways and thereby reducing T-cell exhaustion and improving T-cell survival has been demonstrated previously (9). Julamanee and colleagues extended the knowledge of intracellular costimulatory domains further and showed in their study that a novel B-cell signaling moiety, CD79A/CD40-based CAR, can stimulate both canonical and non-canonical signaling pathways inside the cell and improve the CAR-T cell function (10). In their current study, Ung et al. further explored the downstream mechanisms in CD79A/CD40 based CAR-T cells. The authors reported enrichment of genes known to be associated with T-cell proliferation, interferon signaling, and memory-cell signatures, upregulation of genes related to glycolysis and fatty acid metabolism, and downregulation of T-cell exhaustion in CD79A/CD40 CAR-T cells.

Zhang et al. advanced the research on targeting gp350, an envelope protein of Epstein Barr Virus (EBV) detected in 25% of biopsies from nasopharyngeal carcinoma (NPC) and developed gp350.CAR-T cells. The authors reported preclinical characterization of gp350.CAR-T cells that were produced under good manufacturing practices and noted the applicability of the product for NPC. In another study, Swan et al. aimed to address multiple challenges of CAR-T cells including improving CAR-T cell persistence, tumor penetration, tumor heterogeneity, and possible inhibition of the endogenous immune system due to lymphodepletion by chemotherapy or radiation. The authors developed CAR-T cells that secreted IL7, a cytokine well known to promote T-cell survival and proliferation alone or along with Fms-like tyrosine kinase receptor 3 ligand (Flt3L), a cytokine known to promote DC differentiation, expansion, and survival. They also characterized the activity of the CAR-T cells in a glioblastoma model.

Potez et al. aimed to address the recurrence or relapse of glioblastoma multiforme (GBM) attributed to glioma stem cells (GSCs) and reported the characterization of CAR-T cells targeting GSMs. In their study, researchers combined two previously identified (11), 7-amino acid length peptides that specifically bind to GSCs using *in vitro* and *in vivo* phage display, biopanning through a flexible linker peptide to develop CAR-T cells targeting GSCs. The combined peptide with 29 amino acids was used in place of scFv as an antigen binding domain in the study. Authors reported that the peptide-based CAR-T cells (E-28t28z-tCD34) had significantly higher IFN-Υ secretion when co-cultured with GSCs compared to differentiated glioma cells and showed that N-cadherin was the likely ligand on tumor cells that bound to the CAR.

Solid tumors are considered resistant to CAR-T cells due to the immunosuppressive tumor microenvironment that favors the expression of inhibitory immune checkpoints on effector T-cells such as T cell immunoreceptor with immunoglobulin and ITIM domain (TIGIT) (12). Yang et al. aimed to address the expression of inhibitory immune checkpoints through anti-mesothelin CAR-T cells that constitutively produce TIGIT-blocking single-chain variable fragments. In their article, authors characterized the activity of anti-mesothelin CAR-T cells that produced anti-TIGIT scFvs constitutively using *in vitro* and *in vivo* experiments and reported that the self-delivery of anti-TIGIT scFvs resulted in enhanced infiltration and activity of CAR-T cells in the TME.

Treatment-related adverse events and subsequent hospitalizations are a concern for CAR-T cell therapy. The retrospective observational cohort study by Lipe et al. analyzed the emergency department (ED) visits of patients after receiving CAR-T cell therapy and the association with survival outcomes. Authors reported that patients with ED visits within 14 days of CAR-T cell treatment had significantly better survival outcomes compared to patients with ED visits after 14 days of CAR-T cell treatment; they explained that earlier ED visits were mainly due to an inflammatory response to CAR-T cell therapy possibly resulting in better survival outcomes and the later ED visits were likely due to disease progression resulting in worse survival. In the case report and literature review study by Nogués-Castell et al., authors presented the case history of a patient with plasma cell leukemia (PCL) who achieved complete response after CAR-T cell therapy and later developed orbital tumors consistent with plasmacytoma in both eyes.

Adoptive cell therapy using T-cells expressing transgenic T-cell receptors (TCR-T cells) is an alternative to CAR-T cell therapy and is believed to have advantages over CAR-T cells for solid tumors due to their ability to target intracellular antigens in an MHC-dependent manner. Mercier-Letondal et al. studied engineered TCR-T cells and characterized the phenotype and functional features of atypical TCR-T cells by generating mismatched MHC II-restricted TCR/CD8-expressing T cells and cytotoxic CD4+ T cells against HPV16-derived epitope.

In summary, the articles published as part of the Research Topic covered broad areas of research in cell therapy, ranging from binding domains and intracellular signaling domains and insights into TCR-T cell engineering to real-world outcomes of CAR-T cells. Additional follow-up studies in animal models as well as phase 1 clinical studies may confirm the safety and efficacy of newly developed cell therapies.

## Author contributions

AR: Writing – original draft, Writing – review & editing.
